# Virological Failure After Switch to Long-Acting Cabotegravir and Rilpivirine Injectable Therapy: An In-depth Analysis

**DOI:** 10.1093/cid/ciae016

**Published:** 2024-01-11

**Authors:** Berend J van Welzen, Steven F L Van Lelyveld, Gerjanne Ter Beest, Jet H Gisolf, Suzanne E Geerlings, Jan M Prins, Gitte Van Twillert, Cees Van Nieuwkoop, Marc Van der Valk, David Burger, Annemarie M J Wensing

**Affiliations:** Department of Infectious Diseases, University Medical Center Utrecht, Utrecht, The Netherlands; Department of Internal Medicine, Spaarne Gasthuis, Hoofddorp/Haarlem, The Netherlands; Department of Internal Medicine, Rijnstate Hospital, Arnhem, The Netherlands; Department of Internal Medicine, Rijnstate Hospital, Arnhem, The Netherlands; Infectious Diseases, Department of Internal Medicine, Amsterdam University Medical Center, University of Amsterdam, Amsterdam, The Netherlands; Infectious Diseases, Department of Internal Medicine, Amsterdam University Medical Center, University of Amsterdam, Amsterdam, The Netherlands; Department of Internal Medicine, Noordwest Ziekenhuisgroep, Alkmaar, The Netherlands; Department of Internal Medicine, Haga Teaching Hospital, The Hague, The Netherlands; Infectious Diseases, Department of Internal Medicine, Amsterdam University Medical Center, University of Amsterdam, Amsterdam, The Netherlands; Stichting hiv Monitoring, Amsterdam, The Netherlands; Department of Pharmacy, Institute of Medical Innovation, Radboud University Medical Center, Nijmegen, The Netherlands; Translational Virology, Department of Medical Microbiology, University Medical Center Utrecht, Utrecht, The Netherlands; Ezintsha, Department of Health, University of the Witwatersrand, Johannesburg, South Africa

**Keywords:** long-acting, cabotegravir, rilpivirine, injectables, virological failure

## Abstract

**Background:**

Long-acting (LA) injectable therapy with cabotegravir (CAB) and rilpivirine (RPV) is currently used as maintenance treatment for human immunodeficiency virus type 1, and has a low risk for virological failure (VF). Although the risk is low, the circumstances and impact of VF in the real-world setting merit further evaluation.

**Methods:**

We performed an in-depth clinical, virological, and pharmacokinetic analysis on the reasons behind and the impact of VF during LA CAB/RPV therapy in 5 cases from the Netherlands. Genotypic resistance testing was performed after the occurrence of VF, and drug plasma (trough) concentrations were measured after VF was established and on any other samples to assess on-treatment drug levels. CAB and RPV drug levels that were below the first quartile of the population cutoff (≤Q1) were considered to be low.

**Results:**

Five cases who were eligible for LA CAB/RPV experienced VF despite a low predicted risk at baseline. Genotypic resistance testing revealed extensive selection of nonnucleoside reverse transcriptase inhibitor–associated mutations in all cases, and integrase strand transfer inhibitor mutations in 4 cases. All cases displayed low drug levels of either CAB, RPV, or both during the treatment course, likely contributing to the occurrence of VF. In 3 cases, we were able to identify the potential mechanisms behind these low drug levels.

**Conclusions:**

This is the first in-depth multiple case analysis of VF on LA CAB/RPV therapy in a real-world setting. Our observations stress the need to be aware for (evolving) risk factors and the yield of a comprehensive clinical, virological, and pharmacokinetic approach in case of failure.

## Teaser

Long-acting injectable therapy with cabotegravir and rilpivirine is used as maintenance treatment for HIV-1 and has a low risk for virological failure. We present the first in-depth analysis of 5 cases of real-world virological failure on cabotegravir/rilpivirine therapy.

In December 2020, the first complete long-acting (LA) injectable antiretroviral therapy (ART) combination consisting of the second-generation integrase strand transfer inhibitor (INSTI) cabotegravir (CAB) and the nonnucleoside reverse transcriptase inhibitor (NNRTI) rilpivirine (RPV) was approved by the European Medicines Agency for maintenance treatment in people with human immunodeficiency virus type 1 (HIV-1) [1]. This 2-drug ART regimen is now recommended in the treatment guidelines for people with HIV-1 (PWH) who are virologically suppressed on a stable antiretroviral regimen without present or past evidence of viral resistance to, and no prior virological failure (VF) with, agents of the NNRTI and INSTI class, and no active hepatitis B virus (HBV) infection [2, 3].

The virological efficacy of LA CAB/RPV was assessed in multiple randomized clinical trials, showing noninferiority compared to daily oral INSTI-containing ART through 152 weeks and a low risk (1.2%) for confirmed VF, defined as 2 consecutive HIV-1 RNA measurements (viral loads [VLs]) >200 copies/mL [4–12]. So far, limited data from few real-world cohorts have been published, confirming high efficacy rates as observed in the trials [13–15]. VF did also occur in these real-world cohorts, but the description and analyses of these cases are limited.

Although its occurrence is rare, VF during LA CAB/RPV merits further evaluation: It causes great uncertainty in both PWH and healthcare providers on the reasons behind the failure and can have significant impact on future treatment options due to the selection of resistance-associated mutations (RAMs), which is a rare event in the current era of oral second-generation INSTIs [16]. Here, we present the clinical, virological, and pharmacokinetic analyses of 5 cases with VF on LA CAB/RPV therapy.

## METHODS

Five cases of VF leading to discontinuation of LA CAB/RPV therapy were reported from different HIV treatment centers in the Netherlands. Consent for publication was obtained from all individuals. We defined the moment of VF when physicians decided to discontinue LA CAB/RPV due to a detectable viremia. In the European Union only, the bimonthly dosing (CAB 600 mg and RPV 900 mg) after the initial loading phase is available, but the oral lead-in is optional. The injections may be administered in a time period up to 7 days before or after the due date and, in case this is not possible, bridging with oral CAB/RPV is recommended [17].

We reviewed the clinical history, and a comprehensive virological and pharmacokinetic assessment was performed in all cases after VF had been established. Genotypic resistance testing was performed and the HIV-1 sequence analysis was interpreted with International Antiviral Society USA (IAS-USA) resistance tables and Comet subtype tool [18, 19]. Pharmacokinetic evaluation included measurement of drug plasma concentrations, which was performed with liquid chromatography–mass spectrometry on ethylenediaminetetraacetic acid plasma at or after the moment VF was established and, if available, on other samples drawn to assess on-treatment trough levels [20]. Expected drug concentrations for the bimonthly injectable administration are as follows: for RPV, in a steady state the mean population trough concentration is 0.066 mg/L and the first quartile (Q1) is 0.032 mg/L; the protein binding adjusted inhibitory concentration (PA-IC_90_) is 0.012 mg/L. For CAB, in a steady state the mean population trough concentration is 1.6 mg/L and the Q1 is 1.12 mg/L; the PA-IC_90_ for CAB is 0.166 mg/L [21]. The retrospectively collected drug levels were interpreted in line with recommendations of the French National AIDS Research Agency–Emerging Infectious Diseases (ANRS-MIE) [21] and the cutoffs used in earlier studies [22], which consider drug trough levels ≤Q1 as an alert threshold.

## RESULTS

**Figure 1.**
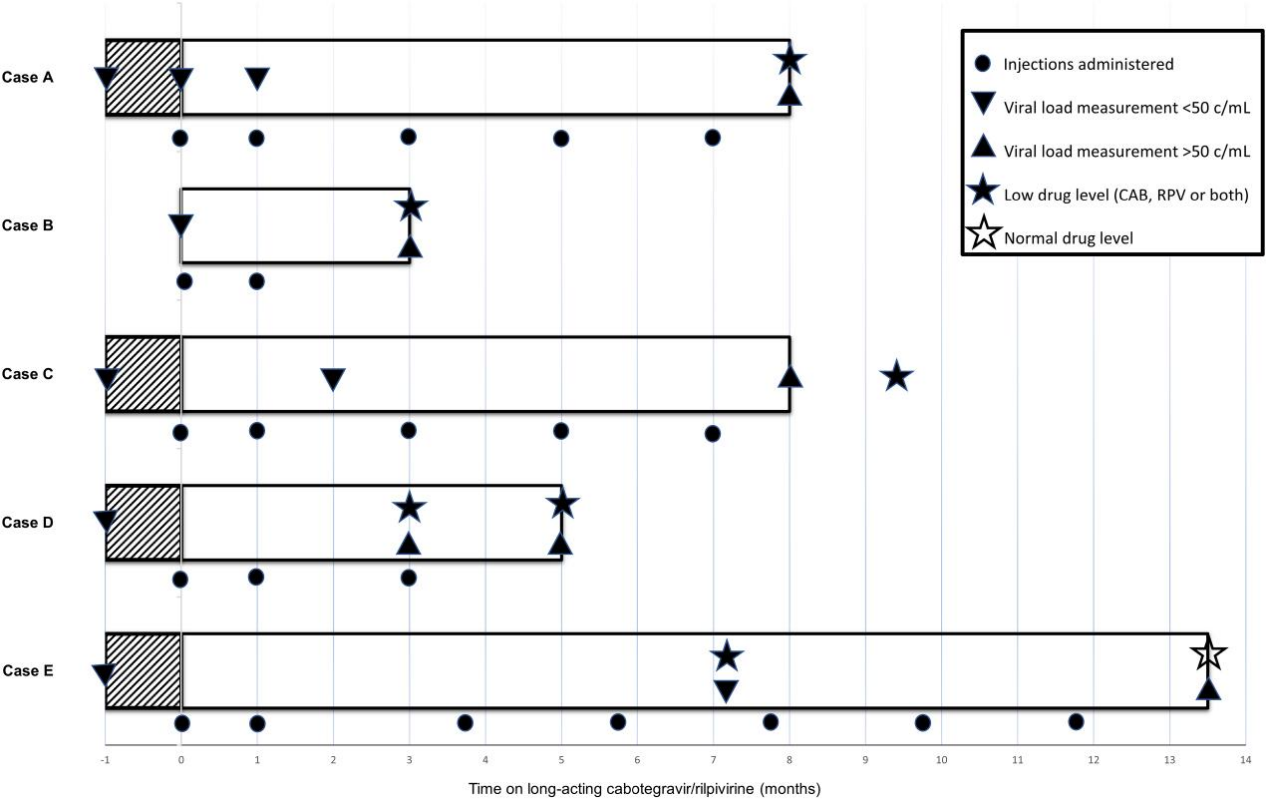
Cabotegravir (CAB) plus rilpivirine (RPV) treatment trajectories of the 5 cases from start to discontinuation. The symbols illustrate the timing of injection administration, viral load (copies/mL), and drug level measurement. The shaded area represents the oral lead-in period of 28 days.

All 5 cases experiencing VF were eligible for LA CAB/RPV at the pretreatment assessment; all had an undetectable HIV VL, and none had a history of VF or a concurrent HBV infection. Baseline resistance testing for reverse transcriptase (RT) RAMs was available in all cases, but baseline integrase gene sequencing had not been performed. All injections were administered by certified healthcare providers, either in the hospital or in the home setting. Most of the events of VF occurred in the first months of therapy. The case characteristics at baseline and outcomes at VF are reported in Tables 1 and 2, respectively. The time course is displayed in Figure 1 and an overview of ART history is provided in Supplementary Table 1.

**Table 1.** Clinical and Virological Characteristics of the Cases

Case	Age at VF, y	Sex	HIV Subtype	RT Domain Genotyping at Baseline	Nadir CD4^+^ Cell Count/μL	Pre-ART VL, Copies/mL	Start of ART	Co-medication During LA CAB/RPV	BMI Prior to LA CAB/RPV, kg/m^2^	BMI at Moment of VF, kg/m^2^	ART Immediately Prior to LA CAB/RPV	Oral Lead-in	Time on LA CAB/RPV
A	38	Transgender woman	B & D recombination	WT	270	1.1 × 10^5^	2015	Quetiapine Estradiol Triptorelin Paroxetine	29.0	33.5	TDF/FTC + DTG	Yes	8 mo
B	49	Cisgender man	B	179D	50	7.5 × 10^6^	2013	Lorazepam Folic acid Naproxen	28.0	28.0	TAF/FTC + DTG	No	3 mo
C	50	Cisgender man	B	WT	20	1.8 × 10^5^	2014	…	31.5	32.7	DTG/3TC	Yes	8 mo
D	56	Cisgender man	B	WT	90	2.2 × 10^5^	2004	Bupropion	27.0	27.1	DTG + DRV/c	Yes	5 mo
E	43	Cisgender woman	B	WT	330	3.8 × 10^4^	2019	Metronidazole Calcium carbonate Vitamin D	45.5	45.5	TAF/FTC/DRV/c	Yes	13 mo

Abbreviations: ART, antiretroviral therapy; BMI, body mass index; DRV/c, darunavir/cobicistat; DTG, dolutegravir; FTC, emtricitabine; HIV, human immunodeficiency virus; LA CAB/RPV, long-acting cabotegravir/rilpivirine; RT, reverse transcriptase; TAF, tenofovir alafenamide; TDF, tenofovir disoproxil; VF, virological failure; VL, viral load; WT, wildtype; 3TC, lamivudine.

**Table 2.** Results of the Virological and Pharmacokinetic Analyses

Case	Moment of VF	VL at Virological Failure, Copies/mL	INSTI RAMs	Post-VF INSTI Resistance^a^	NNRTI RAMs	Post-VF NNRTI Resistance^a^	CAB Trough Levels (mg/L) and Time After Initiation of LA CAB/RPV	RPV Trough Levels (mg/L) and Time After Initiation of LA CAB/RPV
A	March 2023	1.5 × 10^4^	138K, 148R	BIC: Intermediate CAB: High-level DTG: Intermediate EVG: High-level RAL: High-level	101E, 138K	DOR: Low-level EFV: Low-level ETV: Low-level NVP: Intermediate RPV: High-level	2.5* (+8 mo)	0.005* (+8 mo)
B	July 2022	8.3 × 10^6^	None	BIC: Susceptible CAB: Susceptible DTG: Susceptible EVG: Susceptible RAL: Susceptible	101E, 103R, 179D, 181C, 189I	DOR: Low-level EFV: High-level ETV: High-level NVP: High-level RPV: High-level	0.42 (+3 mo)	0.033 (+3 mo)
C	May 2023	9.4 × 10^3^	140S, 148R	BIC: Intermediate CAB: High-level DTG: Intermediate EVG: High-level RAL: High-level	101E	DOR: Low-level EFV: Low-level ETV: Low-level NVP: Intermediate RPV: Intermediate	0.89** (+9.5 mo)	0.038** (+9.5 mo)
D	December 2022	6.1 × 10^5^	155H	BIC: Potential low-level CAB: Low-level DTG: Potential low-level EVG: High-level RAL: High-level	101E, 138K, 230L	DOR: High-level EFV: High-level ETV: High-level NVP: Intermediate RPV: High-level	(1) 0.51 (+3 mo) (2) 0.26 (+5 mo)	(1) 0.013 (+3 mo) (2) 0.020 (+5 mo)
E	March 2023	6.2 × 10^2^	138K, 148K	BIC: Intermediate CAB: High-level DTG: Intermediate EVG: High-level RAL: High-level	90I, 106A, 138K	DOR: High-level EFV: Intermediate ETV: Potential low-level NVP: High-level RPV: Intermediate	(1) 1.4*** (+7 mo) (2) 1.2 (+13.5 mo)	(1) 0.021*** (+7 mo) (2) 0.69 (+13.5 mo)

NNRTI RAMs that were present at baseline are underlined. Drug levels ≤Q1 are underlined. All drug levels were measured 8 weeks after the previous LA CAB/RPV administration except as marked: *4 weeks after previous administration; **10 weeks after previous administration; ***6 weeks after previous administration.

Abbreviations: BIC, bictegravir; CAB, cabotegravir; DOR, doravirine; DTG, dolutegravir; EFV, efavirenz; ETV, etravirine; EVG, elvitegravir; HIV, human immunodeficiency virus; INSTI, integrase strand transfer inhibitor; LA, long-acting; NNRTI, nonnucleoside reverse transcriptase inhibitor; NVP, nevirapine; RAL, raltegravir; RAM, resistance-associated mutation; RPV, rilpivirine; VF, virological failure; VL, viral load.

^a^Stanford University HIV Drug Resistance Database.

### Case A

A 38-year-old transgender woman was diagnosed with HIV in 2015 and subsequently started on ART, achieving rapid virological suppression. In 2019, she started the gender transition program, using feminizing hormone therapy.

In 2022, LA CAB/RPV was initiated: After 5 injection series, all within the target range, VF was established in March 2023. Resistance testing revealed both INSTI and NNRTI RAMs. Pharmacokinetic analysis on a sample drawn 4 weeks after the last injection series showed normal CAB (2.5 mg/L) but very low RPV concentration (0.005 mg/L). This could be the result of the fact that although the patient’s body mass index (BMI) increased from 29.0 to 33.5 kg/m^2^ during the treatment course, the needle length was not adjusted.

ART was initially switched to INSTI-based triple therapy plus maraviroc and later to doravirine, tenofovir disoproxil, and lamivudine plus maraviroc; a protease inhibitor–based regimen was not possible due to drug–drug interactions. Virologic resuppression was achieved after 3 months.

### Case B

A 49-year-old cisgender man was diagnosed with HIV in 2013 while receiving treatment for a diffuse large cell B-cell lymphoma. Baseline genotyping revealed no relevant resistance mutations other than a non-IAS-USA–listed 179D polymorphism in the RT domain, which was not expected to significantly impact NNRTI susceptibility. After the initiation of INSTI-based ART, virological suppression was achieved within 6 months.

In April 2022, he initiated LA CAB/RPV without an oral lead-in. Three months after the initiation, VF was established with a high VL. Subsequent resistance testing showed extensive resistance for NNRTIs, but no RAMs in the integrase domain. Pharmacokinetic analysis was performed on the same sample showing a RPV trough concentration just above Q1 (0.036 mg/L) and a low CAB trough concentration (0.42 mg/L). These findings were suggestive for a problem with the administration of 1 of the CAB injections during the loading phase. However, neither the patient nor the involved healthcare providers recalled any abnormalities. All injections had been administered within the prescribed time window. Pending the resistance results, treatment with tenofovir alafenamide (TAF), emtricitabine (FTC), and darunavir/cobicistat (DRV/c) was initiated and eventually switched back to TAF/FTC plus dolutegravir (DTG). Virologic resuppression was achieved in 6 months.

### Case C

A 49-year-old cisgender man initiated LA CAB/RPV after being on ART since 2014. At baseline, there were no risk factors for failure other than a BMI of 32 kg/m^2^. As the body fat was mainly localized in the abdominal area, needle length was not adjusted by the treatment team. He received 5 injection series and all were in the advised time range; the VL 1 month after the loading phase was <50 copies/mL. In May 2023, viral breakthrough occurred with selection of extensive INSTI RAMs, leading to intermediate-to-high resistance for all INSTIs and 1 NNRTI RAM leading to low-to-intermediate NNRTI resistance. Ten weeks after the last injection series, drug levels were measured and showed low CAB (0.89 mg/L) and adequate RPV levels (0.038 mg/L). No reason for these pharmacokinetic findings could be identified. ART was switched to TAF/FTC/DRV/c, resulting in an undetectable VL in November 2023.

### Case D

A 56-year-old cisgender man was diagnosed with HIV in 2004 and started on ART. During follow-up he received multiple antiretroviral regimens, including NNRTI- and INSTI-containing ART, without any events of VF. After being on a stable nucleos(t)ide-sparing regimen (DTG/DRV/c) because of a polyneuropathy, he switched to CAB/RPV in the summer of 2022. Four months later, a low-level viremia (260 copies/mL) was found but LA CAB/RPV was continued. By the next injection series in December, the VL had increased to 610 000 copies/mL, leading to therapy discontinuation. Virological analyses revealed significant INSTI and NNRTI resistance. Retrospective pharmacokinetic analyses found consistently low CAB and RPV levels: During the episode of low-level viremia, the CAB concentration was 0.51 mg/L and RPV concentration was 0.013 mg/L, and at the moment of definitive discontinuation the trough concentrations were 0.26 and 0.02 mg/L, respectively. All injections had been administered in time and no factors for the consistently low CAB and RPV levels could be identified. After VF, ART was switched to TAF/FTC/DRV/c and resuppression was achieved.

### Case E

A 43-year-old cisgender woman commenced ART (TAF/FTC/bictegravir) in 2019. Her medical history was remarkable for obesity, with a BMI of 45.5 kg/m^2^ and a chronic prosthetic joint infection, for which she used metronidazole as suppressive antibiotic therapy during CAB/RPV initiation. After oral lead-in, LA CAB/RPV was started in February 2022 with the use of long injection needles (21 gauge, 51 mm). The administration of the first injections series was unremarkable, but the third injection series was administered 11 weeks and 2 days after the second series due to a stay abroad. She did not use oral bridging to cover for this delay. By the end of August 2022 the VL was 45 copies/mL. The subsequent injections series were all within the advised time range. No follow-up VLs were performed till March 2023, when viral breakthrough was established (VL, 620 copies/mL) and the selection of both INSTI and NNRTI RAMs. Retrospective trough level measurement was performed on the samples of August 2022 and March 2023: The first sample showed normal CAB (1.4 mg/L) and low RPV (0.021 mg/L) levels, possibly due to the delayed third injection series. The second sample at VF showed normal levels for both CAB and RPV. In this case, it seems likely that the prolonged dosing interval between the second and third series contributed to the development of VF. After the occurrence of VF, she decided not to restart oral ART.

## DISCUSSION

In this analysis, we studied 5 cases of VF on LA CAB/RPV in PWH who were eligible for this treatment and had a low risk for failure at baseline. All selected extensive NNRTI and/or INSTI RAMs at the time of VF. Pharmacokinetic analysis revealed low drug levels (ie, below Q1) during the treatment course in all cases. The occurrence of VF after lengthy virological suppression on oral ART had a profound impact on the involved PWH and healthcare providers.

The reported occurrence of VF in LA CAB/RPV trials is low—only 26 cases in 2105 trial participants (1.2%) with most events in the first year of therapy [4–12]. In a post hoc analysis of pooled trial data that included 23 cases [23]—without the 3 recently reported cases [12]—the baseline characteristics subtype A6/A1, the presence of RPV RAMs, and a BMI ≥30 kg/m^2^ were associated with higher odds for confirmed VF. PWH with the presence of 2 or more of these factors were at increased risk for VF. Therefore, it is advised to consider these factors before initiating LA CAB/RPV to minimize VF risk. In these pooled trial data, the risk for failure was low for those with zero risk factors (0.9% [4/970]) and 1 risk factor (2% [8/404]) present at baseline [23] . In our analysis, 3 of the 5 cases had no known risk factors at baseline; the others had only high BMI. Although the absolute risk for VF is low, our case descriptions illustrate that it is important not only to consider these baseline risk factors, such as in case C, but also to continuously reconsider evolving risks after LA CAB/RPV initiation: For case A, needle length adjustment was indicated as she gained a substantial amount of weight, and for case E the belated third injection series should have given rise to more intensified monitoring.

Resistance testing at the moment of VF showed that all cases acquired NNRTI RAMs and in 4 cases there was also selection of INSTI RAMs, leading to a nearly complete loss of the NNRTIs and decreased susceptibility within the INSTI class. Only case B retained full INSTI susceptibility; in most of the remaining cases, twice-daily dosed DTG will be needed to overcome INSTI resistance [18]. Although the pattern of widespread and fast acquisition of INSTI RAMs in this analysis is in line with the trials [24], its occurrence remains remarkable. Second-generation INSTIs have a high genetic barrier for resistance, and the selection of INSTI RAMs in persons without a history of VF is rare [16]. In a recent publication studying individuals with treatment failure on a DTG-based regimen, 89.5% of them harbored viral strains that remained fully susceptible to second-generation INSTI and 85% to all INSTIs [25]. In that sense, the rapid loss of CAB and first-generation INSTIs as treatment options is striking, and this risk is explicitly mentioned in the IAS-USA guidelines [3] but not in the European guidelines [2]. This phenomena is likely to be multicausal; it has been postulated that CAB-containing regimens have a relatively low genetic barrier for resistance when combined with another low genetic barrier antiretroviral such as RPV [26]. CAB appears to select RAMs more easily than DTG in case of failure, with these RAMs having a profound impact on INSTI susceptibility. In addition, in case of incorrect administration, the long dosing interval leads to longer exposure to subtherapeutic drug levels than in oral therapy, increasing the risk for resistance development. The high risk for the acquisition of NNRTI and INSTI RAMs in the rare event of virological failure raises the question to what degree this risk for resistance selection can be accepted, especially as the other guideline-recommended switch strategies do not lead to the potential loss of 2 antiretroviral drug classes [27]. This question is even more relevant when LA CAB/RPV becomes a treatment option in high-endemic, low- and middle-income countries, in which frequent VL monitoring is more challenging and therefore the occurrence of VF and selection of RAMs can go unnoticed for a longer time period [28]. In high-income countries, a potential strategy to mitigate this risk would include even more intensive VL monitoring (eg, every 3–4 months) for the early detection of VF in selected cases—for example, those with risk factors at baseline or in case of belated injection administration. However, intensified monitoring can be challenging in those having difficulties with visiting the clinic and will inevitably lead to finding low-level viremias [29], potentially triggering treatment alterations that are unnecessary in most cases.

In addition to the virological evaluation, we reported pharmacokinetic measurements and found that in 4 cases low CAB and/or RPV levels (≤Q1) were present at the moment of failure, and in 1 case RPV level was low during the treatment course but not at the moment of VF. The pattern of the low drug levels was variable: 1 case had both low CAB and RPV levels, and the other cases displayed either low CAB or RPV levels. Since drug levels were mainly available at the moment of failure, in some cases it remains unclear whether the observed levels are due to a single erroneous administration, or that the low drug levels have a more structural nature. Only for case D did we find consistently low trough levels at 2 timepoints, suggesting a more structural causative mechanism rather than a problem with 1 injection, which was more likely in cases B and E.

The number of factors known for contributing to low CAB and/or RPV levels after intramuscular administration is limited, but recent real-world data have shown significant inter- and intra-individual variability and have also shown that CAB concentrations are substantially lower than previously reported, albeit sufficient to ensure sustained virological suppression [30]. A factor that could contribute to low CAB levels early in the treatment course is the lack of an oral lead-in, which was applicable in case B, although this has not been identified as a predictor for VF [31]. The relationship between high BMI and drug levels remains a topic of debate; although a higher BMI was independently associated with VF, another analysis on pharmacokinetic trial data found no clear association between BMI ≥30 kg/m^2^ and drug levels [32]. It has also been implicated that a body composition in which the adipose tissue is mainly localized in the pelvis and hip area poses an increased risk for low drug levels and subsequent VF, even in case the BMI is <30 kg/m^2^ [33]. These findings emphasize the need for a better understanding of inter- and intra-individual pharmacokinetic variability, mechanisms behind low drug levels, and the relationship with VF. In addition, healthcare providers need to remain alert on the correct administration technique.

Our analysis shows that it can be useful to measure drug levels in case of VF to identify reasons behind VF, but the role for drug level measurement as part of regular care still needs to be defined. In the first multivariable analysis by Cutrell et al, RPV levels ≤Q1 at week 8 were present in 9 of 13 PWH with VF, and 8 of 13 displayed CAB levels ≤Q1 [22]. The role of low drug levels in relation to VF is further supported by the observation from the trials that all 7 PWH, who experienced VF in absence of any baseline risk factors in the trials, displayed low drug levels [12, 23]. In addition, the recently published prediction model found that 44-week CAB and RPV levels are associated with a higher incidence rate ratio for VF: 5.99 (95% confidence interval [CI], 1.94–18.5; *P* = .019) and 4.16 (95% CI, 1.04–16.7; *P* = .041) per 1 log_2_ unit decrease, respectively [23]. Although the authors of the article argue that adding drug level measurement to the prediction rule is complex and has limited additional value, we believe that measurement of an early, steady-state trough level at week 8 can be helpful to identify high-risk patients (ie, those with 1 risk factor at baseline). In the future, drug level measurement might also be used to adjust dosage in case of low drug levels.

In conclusion, this is the first in-depth case analysis of VF in multiple PWH receiving LA CAB/RPV as maintenance therapy in a real-world setting, showing low drug levels and the selection of extensive resistance in all cases. Our findings call for awareness of the possibility of VF due to evolving factors, as well as a comprehensive clinical, virological, and pharmacokinetic approach in case of failure. Future studies should focus on the reasons for low drug levels and their relationship with VF, and on strategies to limit the rapid loss of ART options in the rare event of failure.

## Supplementary Data

Supplementary materials are available at *Clinical Infectious Diseases* online. Consisting of data provided by the authors to benefit the reader, the posted materials are not copyedited and are the sole responsibility of the authors, so questions or comments should be addressed to the corresponding author.

## Supplementary Material

ciae016_Supplementary_Data

## Notes

***Author Contributions.*** B. J. W. drafted the manuscript in close collaboration with D. B. and A. M. J. W. All authors interpreted the findings, were involved in the drafting and review of the manuscript, and approved the final version.

***Acknowledgments.*** The authors thank the involved patients for their permission for publication.
