# Supplementary material for: Virological Failure After Switch to Long-Acting Cabotegravir and Rilpivirine Injectable Therapy: An In-depth Analysis
Source: Clin Infect Dis. 2024 Jan 11;79(1):189–95. doi: 10.1093/cid/ciae016 (PMC11259215; doi:10.1093/cid/ciae016)
Supplement: ciae016_Supplementary_Data [file ciae016_supplementary_data.docx]

|  | *Year of treatment initiation/switch* | *Antiretroviral regimen* | *Reason for switch* |
| --- | --- | --- | --- |
| Case A | 2015 | TDF/FTC + DTG | - |
| Case B | 2013  2013  2014  2018  2018 | TDF/FTC + RAL + MVC  TDF/FTC + RAL  TDF/FTC + DTG  BIC/FTC/TAF  TAF/FTC + DTG | -  Simplification after resistance test  Simplification  Simplification  Side effects |
| Case C | 2014  2014  2015  2015  2022 | TDF/FTC + DRV/r + DTG  TDF/FTC + DRV/r  TDF/FTC + DTG  ABC/3TC/DTG  3TC/DTG | -  Simplification after resistance test  Side effects  Simplification  Simplification |
| Case D | 2004  2007  2011  2012  2012  2016 | AZT/3TC + EFV  AZT/3TC + NVP  TDF/FTC + NVP  AZT/3TC + NVP  ABC/3TC + NVP  DRV/c + DTG | -  Side effects  Simplification  Side effects  Simplification  Side effects |
| Case E | 2019  2021 | BIC/FTC/TAF  TAF/FTC/DRV/c | -  Side effects |

Supplementary table 1 – Overview of antiretroviral combinations and reasons for treatment switch. Abbreviations: 3TC – lamivudine, ABC – abacavir, AZT – zidovudine, BIC – bictegravir, DRV/c – darunavir/cobicistat, DRV/r – darunavir/ritonavir DTG – dolutegravir, EFV – Efavirenz, FTC – emtricitabine, MVC – maraviroc, NVP - nevirapine, RAL – raltegravir, TAF – tenofovir alafenamide, TDF – tenofovir disoproxil.
